# A short dasatinib and quercetin treatment is sufficient to reinstate potent adult neuroregenesis in the aged killifish

**DOI:** 10.1038/s41536-023-00304-4

**Published:** 2023-06-16

**Authors:** Jolien Van houcke, Valerie Mariën, Caroline Zandecki, Rajagopal Ayana, Elise Pepermans, Kurt Boonen, Eve Seuntjens, Geert Baggerman, Lutgarde Arckens

**Affiliations:** 1 Laboratory of Neuroplasticity and Neuroproteomics, Department of Biology, KU Leuven, 3000 Leuven, Belgium; 2 Laboratory of Developmental Neurobiology, Department of Biology, KU Leuven, 3000 Leuven, Belgium; 3 Centre for Proteomics, University of Antwerp, 2020 Antwerpen, Belgium; 4 Health Unit, VITO, 2400 Mol, Belgium; 5 KU Leuven Brain Institute, KU Leuven, 3000 Leuven, Belgium

**Keywords:** Regeneration and repair in the nervous system, Adult neurogenesis, Ageing, Neural stem cells, Preclinical research

## Abstract

The young African turquoise killifish has a high regenerative capacity, but loses it with advancing age, adopting several aspects of the limited form of mammalian regeneration. We deployed a proteomic strategy to identify pathways that underpin the loss of regenerative power caused by aging. Cellular senescence stood out as a potential brake on successful neurorepair. We applied the senolytic cocktail Dasatinib and Quercetin (D + Q) to test clearance of chronic senescent cells from the aged killifish central nervous system (CNS) as well as rebooting the neurogenic output. Our results show that the entire aged killifish telencephalon holds a very high senescent cell burden, including the parenchyma and the neurogenic niches, which could be diminished by a short-term, late-onset D + Q treatment. Reactive proliferation of non-glial progenitors increased substantially and lead to restorative neurogenesis after traumatic brain injury. Our results provide a cellular mechanism for age-related regeneration resilience and a proof-of-concept of a potential therapy to revive the neurogenic potential in an already aged or diseased CNS.

## Introduction

The African turquoise killifish (*N. furzeri*) has found its place in between short-living invertebrate (*C. elegans, D. melanogaster*) and long-living vertebrate (*M. musculus, D. rerio*) biogerontology models. With a lifespan ranging from four to six months, the killifish facilitates studying aging mechanisms within a reasonable timeframe and within a vertebrate physiology context^1–3^. A sequenced and annotated genome is available for *N. furzeri*^1,4^ which only recently starts to facilitate genome engineering^5^, as well as transcriptomic^6–12^ and proteomic studies^13^. We and others have investigated aging of the killifish central nervous system (CNS) and have found remarkable resemblance to human CNS aging, including an age-related decline in neurogenesis, cognition and visual acuity, spontaneous neurodegeneration and stem cell exhaustion^14–19^. Like other teleosts, killifish have a great capacity for (neuro)regeneration^20^. Yet, we discovered that in killifish, aging induces the limited neurorepair capacities of mammals. These include low reactive proliferation of progenitors, low generation of newborn neurons, high inflammation and permanent glial scarring of the injured aged telencephalon^17^. The disparity in regeneration capacity from successful to unsuccessful with age, makes the killifish a unique preclinical model to find or validate new neuroreparative strategies that would work in the aged injured or diseased brain. Such strategies are desperately needed for patients suffering from age-related neuropathology in which the aging brain state is a critical factor, i.e. Alzheimer’s, Parkinson’s and traumatic brain injury (TBI).

Current knowledge about aging-associated cellular senescence is mostly gained from mammalian in vitro and in vivo models, and to a lesser extent form teleost fish^16,21–25^ or invertebrates^26–29^. Senescent cells accumulate with age as a response to multiple stressors such as increasing DNA damage, telomeric attrition and other oncogenic events^30,31^. The damaged cells go into growth arrest via p53/p21^CIP1^ and p16^INK4^/pRb tumor suppressor pathways, alter gene expression and are metabolically active^30,32^. They acquire a senescence-associated secretory phenotype (SASP), consisting of the secretion of proteases, chemokines, growth factors and extracellular matrix components, which alter the microenvironment of the tissue^33^. This SASP can also turn neighboring cells senescent in a process called paracrine senescence^34,35^. With increasing age the immune system is overwhelmed and unable to remove the increasing number of senescent cells^36^. In addition, senescent cells are resistant to apoptosis by using senescent cell anti-apoptotic pathways (SCAPs). As such, senescent cells pile up in the aged brain, changing the tissue environment and impairing tissue homeostasis and repair^33,37,38^.

Here, we have exploited the killifish biogerontology model to discover pathways which underlie the loss of its regenerative abilities with age. We established an age-dependent differential proteomics dataset which identified a high senescent state in the aged killifish forebrain as one of the possible brakes on successful neuroregeneration. Via short-term systemic treatment of aged fish with the senolytic cocktail Dasatinib (D) and Quercetin (Q) we could diminish cellular senescence in the parenchyma as well as in the neurogenic niches of the aged brain. To elucidate if this treatment could improve neurorepair, we assessed the regeneration capacity of aged D + Q-treated killifish after traumatic brain injury. We witnessed an increased number of proliferating non-glial progenitors (NGPs), leading to an increased generation of new mature neurons. Early after TBI, the number of inflammatory microglia or infiltrating macrophages present at the injury site remained unchanged but the microglia and macrophages did adopt a more ramified morphology, indicative of a resting state. Glial scarring was not prevented. Short-term treatment with D + Q thus enhances the regenerative power of the aged brain, as a way to compensate for brain injuries or disease.

## Results

### Proteomic profiling of the aged killifish telencephalon reveals enrichment of cellular senescence

**Fig. 1:**
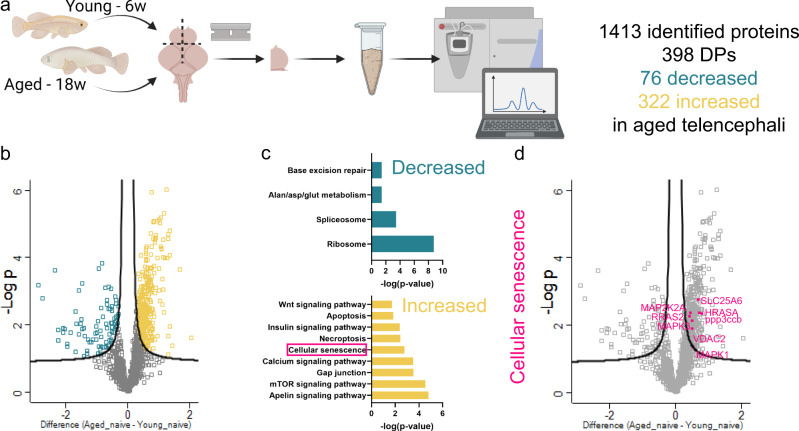
Differential proteomics reveals enrichment of cellular senescence in the aged killifish telencephalon. **a** General workflow of the quantitative proteomics: young and aged killifish brains were extracted; the right hemisphere of the telencephalon quickly dissected; samples homogenized, and proteins extracted and analyzed using a QExactive Quadrupole-Orbitrap MS and label-free quantification. We identified 1413 proteins of which 398 displayed differential levels between young and aged killifish. Of these 398 DPs, 76 proteins displayed a lower level and 322 a higher level in the aged killifish. **b** Volcano plot showing the -log(*p*-value) and difference of the significantly decreased (green) and increased (yellow) proteins due to age (*n* = 6 fish). **c** GO analysis using STRING functional proteins association networks revealed enrichment (*p* < 0,05) of KEGG pathways related to proteins with decreased (green) or increased (yellow) levels due to aging. **d** Volcano plot displaying the DPs that were allocated to the cellular senescence pathway. DP differential protein, GO gene ontology.

To discover pathways that underpin age-related changes in the brain causing impaired neuroregenerative capacity with advancing age, we performed label-free differential proteomics of young and aged killifish. We identified 1413 proteins shared between all samples, of which 398 had differential levels between young and aged telencephalon (Fig. 1a, b, Supplementary Table 1, *n* = 6 fish). Some of the differential proteins (DPs), had already been linked to the aging process of the brain and thus confirmed the quality of our experimental approach^15,39–42^. We measured higher levels of the glial intermediate filament proteins GFAP and Vimentin (VIML), Microtubule-associated protein tau a (MAPTA) and α-Synuclein (SNCA), and lower levels of Microtubule-associated protein tau (MAPT) for example.

We performed pathway enrichment analysis on the 398 DPs using STRING^43^. The KEGG pathways related to lower protein levels were *ribosome*, *spliceosome* and *base excision repair* (Fig. 1c, Supplementary Table 2), pointing towards faulty DNA damage repair mechanisms and altered splicing and translation of transcripts with advancing age. The increased proteins of our dataset were related to well-known aging KEGG pathways, including the *Insulin* and *mTOR signaling* pathways and *cellular senescence* (Fig. 1c, d). Supplementary Table 3 shows the full list of enriched KEGG pathways for increased proteins.

We hypothesized that cellular senescence might be one of the age-related changes that diminish the resilience of an aged brain to brain injuries. Indeed, cellular senescence changes the tissue micro-environment^32,33^, necessary for proper neuroregeneration. We chose to investigate cellular senescence in more depth and aimed to modulate this pathway to improve neuroregeneration.

### Cellular and molecular phenotyping of senescence in the aged killifish telencephalon

**Fig. 2:**
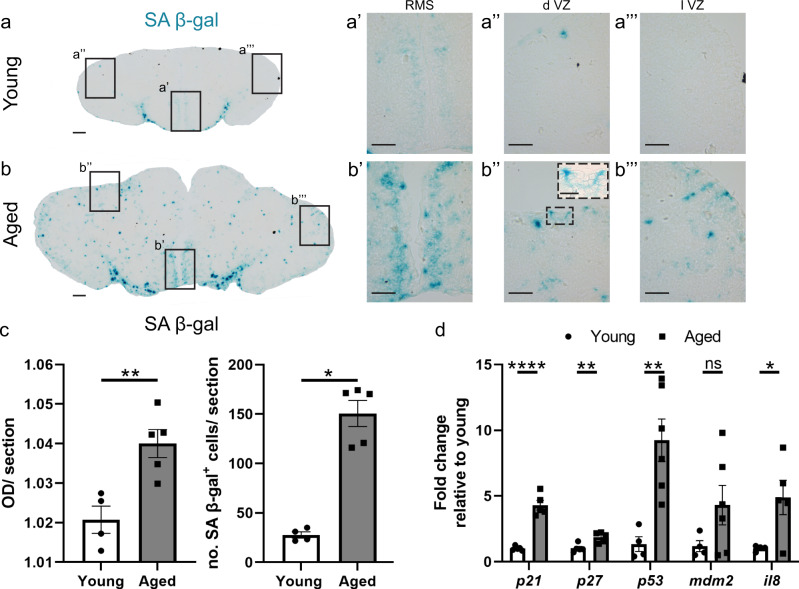
Aging induces a high senescent cell burden in the stem cell niches and parenchyma of the telencephalon. **a**, **b** SA β-gal staining on coronal sections of the telencephalon of young (**a**) and aged (**b**) killifish demonstrates the high amount of senescent cells (blue cells) present in the aged killifish brain. Scale bars: 100 µm. Higher magnifications of the boxed areas in (**a**) and (**b**) illustrate increased cellular senescence in the aged stem cell niches. Scale bars: 50 µm. **b''** Higher magnification of a SA β-gal^+^ progenitor in the d VZ of the aged telencephalon. Scale bar: 20 µm. **c** Optical density (OD) measurement of SA β-gal staining and number of SA β-gal^+^ cells per section of young and aged killifish reveals a higher SA β-gal staining intensity and senescent cell number in aged killifish. *p ≤ 0,05, **p ≤ 0,01; Two-tailed unpaired *t*-test/Mann–Whitney test. Values are mean ± SEM; *n* ≥ 4 fish. **d** Relative expression values of the cell cycle inhibitors *p21* and *p27*, the DNA damage responsive markers *p53* and *mdm2* and the SASP marker *il8*. The expression of *p21*, *p27*, *p53* and *il8* is significantly increased in the aged killifish telencephalon. *p ≤ 0,05, **p ≤ 0,01, ****p ≤ 0.0001; Two-tailed unpaired *t*-test. Values are mean ± SEM; *n* ≥ 4 fish. SA β-gal: senescence-associated β-galactosidase, OD optical density, RMS rostral migratory stream, d VZ dorsal ventricular zone, l VZ lateral ventricular zone. Data in (**c**) are reused from ref. ^17^.

We first validated our proteomics results by checking the senescent state of the aged brain with cellular and molecular assays. Senescence-associated β-galactosidase (SA β-gal) staining is the standard method for detecting senescent cells because of its ease of use. It measures lysosomal β-gal levels at pH 6, which are increased in senescent cells^44,45^. It is a preferred marker when working with non-canonical animal species as it does not require access to any species specific materials (e.g. antibodies). As expected, the aged telencephalon showed a higher level of SA β-gal staining, illustrating a high senescent cell burden (Fig. 2a, b). In the aged fish, we observed SA β-gal^+^ cells in the parenchyma. In addition, the rostral migratory stream (RMS), dorsal ventricular zone (d VZ) and lateral ventricular zone (l VZ) -stem cell niches also held a large number of SA β-gal^+^ senescent cells, presumably progenitors (Fig. 2b). We could distinguish triangular and round cell bodies at the outer border of the VZ, which is typical for radial glia (RGs) and non-glial progenitors (NGPs) in the teleost telencephalon^46^. The *tela choroidea*, the membrane surrounding the ventricle, also showed a large number of SA β-gal^+^ cells, suggesting the presence of senescent ependymal cells^12^. By measuring the intensity of the SA β-gal staining, and by counting the number of SA β-gal^+^ cell bodies in the entire telencephalon, we found a significant increase in SA β-gal in the aged fish. The number of SA β-gal^+^ senescent cells was five times higher in aged killifish compared to young adult killifish (150,5 ± 13,2 versus 27,8 ± 3,2, *p* = 0.016; Fig. 2c). In addition, the aged telencephalon showed increased expression of the cell cycle inhibitors *p21* and *p27*, the damage-responsive markers *p53* and *mdm2* and of the SASP marker *il8* (Fig. 2d)^17^, which are typical hallmarks of cellular senescence.

Taken together, these results corroborate our proteomics strategy and indicate that the aged telencephalon contains an increased load of senescent cells, including progenitors in the neurogenic niches.

### Short-term systemic treatment with senolytics Dasatinib and Quercetin removes senescent cells from the aged killifish telencephalon

Senescent cells secrete proteases, chemokines, growth factors and extracellular matrix components, which alter the microenvironment of the tissue^33^ and might decrease neurogenic potential. For this reason, we addressed whether targeted removal of senescent cells might reestablish a favorable cellular environment for successful neurorepair in the aged injured telencephalon. Senolytic drugs are compounds that target senescent cell SCAPs causing the senescent cell to undergo apoptosis^47^. We chose the senolytic cocktail of Dasatinib and Quercetin (D + Q) to specifically induce apoptosis in senescent cells in the aged killifish brain. Dasatinib is an anti-cancer drug that can target tyrosine kinase-related SCAPs, while quercetin is a natural plant flavonoid that inhibits the BCL-2/BCL-X_L_, PI3K/AKT, and p53/p21/serpine SCAPs^48^. Since we found that the senescence markers *p53* and *p21* showed higher expression in the aged killifish brain (Fig. 2d), the use of quercetin as senolytic was thus straightforward.

To our knowledge, there are currently no studies in teleost fish published using D + Q as senolytic (except for a recent preprint^49^), and as such, we aimed to test whether we could remove senescent cells using D + Q in the very rapidly aging killifish. In most pre-clinical studies, the D + Q cocktail is given every 2 weeks for about 2 months in mice^50,51^. To test the power of the approach, we opted to use a very short treatment, starting at a very old age, when senescent cells are already abundant, to elucidate if such a strategy could alleviate an established senescent cell burden in the aged killifish and re-open a window of increased regenerative potential.

D + Q work synergistic and together they target several pro-survival networks (SCAPs) instead of a single receptor or molecule, which is thought to increase specificity to target senescent cells and reduce off-target effects on healthy cells^52^.

**Fig. 3:**
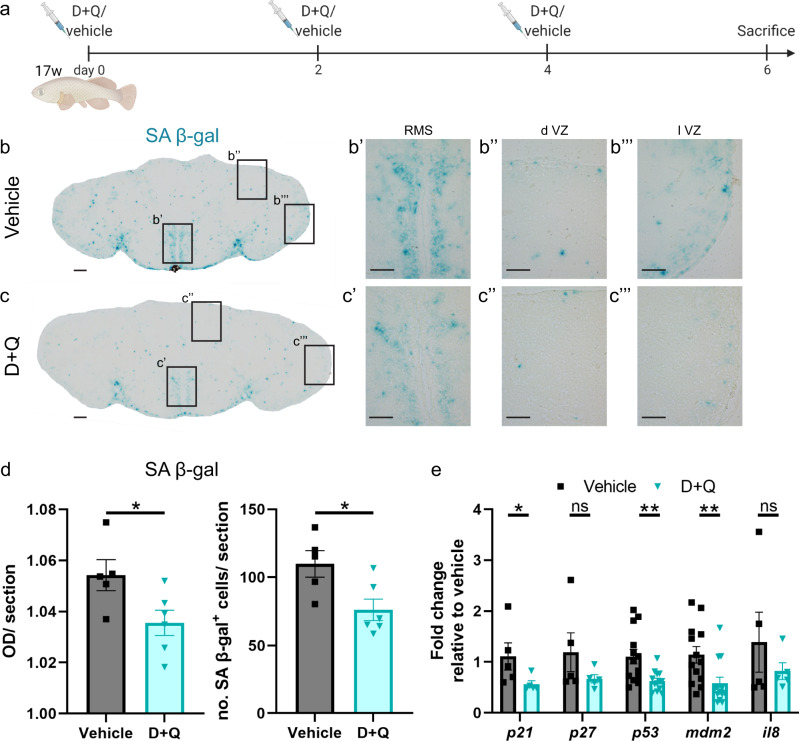
Short-term treatment of the aged killifish with senolytics D **+** Q removes senescent cells from the telencephalon. **a** Experimental setup: 17-week-old aged fish were vehicle-treated or treated with D + Q via 3 consecutive i.p. injections, each separated by 2 days. Samples were made 2 days after the last injection. **b** SA β-gal staining on coronal sections of the telencephalon of aged vehicle- (**b**) and D + Q-treated (**c**) killifish shows diminished SA β-gal activity in D + Q-treated fish. Scale bars: 100 µm. Higher magnifications of the boxed areas in (**b**) and (**c**) illustrate reduced cellular senescence in the stem cell niches of the D + Q-treated killifish. Scale bars: 50 µm. **d** OD measurement of SA β-gal staining and number of SA β-gal^+^ cells per section of aged vehicle- and D + Q-treated killifish reveals a significantly lower SA β-gal staining intensity and decreased number of senescent cells in D + Q-treated killifish. *p ≤ 0,05; Two-tailed unpaired *t*-test. Values are mean ± SEM; *n* ≥ 5 fish. **e** Relative expression values of the cell cycle inhibitors *p21* and *p27*, the DNA damage responsive markers *p53* and *mdm2* and the SASP marker *il8*. The expression of *p21*, *p53* and *mdm2* is significantly decreased in aged D + Q-treated killifish. *p ≤ 0,05, **p ≤ 0,01; Two-tailed unpaired *t*-test/Mann–Whitney test. Values are mean ± SEM; *n* ≥ 4 fish. i.p. intraperitoneal, SA β-gal senescence-associated β-galactosidase. D + Q Dasatinib and Quercetin, OD optical density, RMS rostral migratory stream, d VZ dorsal ventricular zone, l VZ lateral ventricular zone.

We implemented a short-term systemic treatment regimen, with D + Q, via 3 consecutive intraperitoneal (i.p.) injections, each separated by 2 days, in aged fish (Fig. 3a). On day 6 of the experiment, we tested vehicle- and D + Q-treated aged killifish for the manifestation of senescence markers (Fig. 3). D + Q-treatment led to a significant reduction in SA β-gal staining intensity (Fig. 3b–d). Also the number of SA β-gal^+^ cells was reduced with ~30% in D + Q-treated killifish compared to vehicle-treated killifish (76,2 ± 7,8 versus 109,8 ± 9,7, *p* = 0.023, Fig. 3d). The reduction was prominent in the parenchyma and stem cell niches of the telencephalon (Fig. 3b, c), especially in the RMS and the l VZ. Via qPCR we also established a significant reduction in the expression of typical senescence-associated markers in D + Q-treated fish, namely *p21*, *p53* and *mdm2* (Fig. 3e). A considerable reduction in the expression of *p27* and *il8* was also observed (Fig. 3e). Multiple studies have already illustrated the specificity of D + Q to drive senescent cells into apoptosis and report on the increased presence of apoptotic cells shortly after D + Q administration^47,53–60^. Since D and Q have elimination half-lives of <12 h^61,62^, we also probed for the presence of apoptotic cells six h after a single i.p. D + Q injection. Via TUNEL labelling, which detects DNA fragments in early and late apoptotic cells, we detected a fourfold increase in TUNEL^+^ cell number in D + Q- versus vehicle-treated aged fish (16,2 ± 2,2 versus 3,5 ± 1,02, *p* = 0.0009, Supplementary Fig. 1).

Collectively, our data show that a short treatment of three injections with D + Q is sufficient to reduce the senescent cell burden in aged killifish most probably because D + Q induces apoptosis of senescent cells.

### Short treatment with D **+** Q improves neuroregeneration of the aged telencephalon after stab-wound injury

Since we were able to diminish cellular senescence in the aged killifish brain, we questioned if aged D + Q-treated fish have improved neuroregenerative capacities. Using a TBI model, we previously established that aged killifish show diminished progenitor cell proliferation and generation of neurons, and an overall impaired neurorepair in contrast to young adult killifish^17^. In case D + Q reintroduces cellular capacities that typify a younger brain, neurorepair might become successful again.

**Fig. 4:**
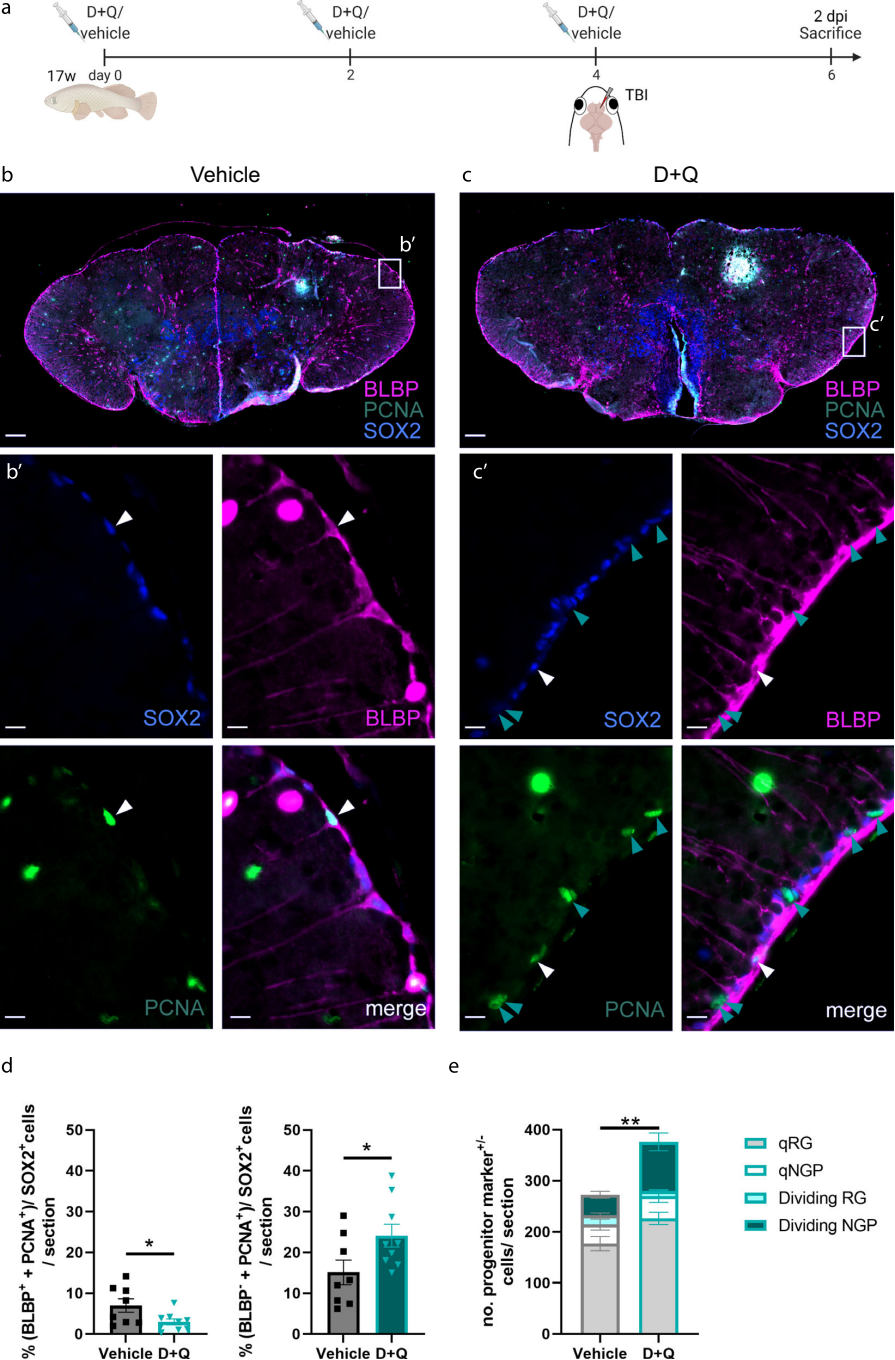
Short-term treatment of the aged killifish with D **+** Q boosts progenitor proliferation after TBI. **a** Experimental setup: 17-week-old aged fish were vehicle-treated or treated with D + Q via 3 consecutive i.p. injections, each separated by 2 days. Together with the last injection, fish received a TBI. Samples were made 2 days after the last injection/TBI (at 2 dpi). The injury size is on average 0.008mm^2^ ± 0.005 (mean ± SD)^17^, but, depending if a blood vessel is pierced, can look smaller or larger on fluorescent images, since blood will non-specifically fluoresce in the green channel. **b**, **c** Triple staining for BLBP (magenta), PCNA (green) and SOX2 (blue) on coronal sections of vehicle- (**b**) and D + Q-treated (**c**) aged killifish at 2 dpi. The injury site is filled with red blood cells that fluoresce in the green channel. Scale bars: 100 µm. **b'**, **c'** Magnification of the boxed areas in (**b**) and (**c**) respectively. White arrowheads depict triple-positive BLBP^+^ SOX2^+^ PCNA^+^ dividing RGs, while turquoise arrowheads mark double-positive BLBP^-^ SOX2^+^ PCNA^+^ dividing NGPs. More dividing NGPs are noticed in D + Q-treated fish in the VZ. Scale bars: 10 µm. **d** Proportion of BLBP^+^ SOX2^+^ PCNA^+^ dividing RG and BLBP^-^ SOX2^+^ PCNA^+^ dividing NGPs over all SOX2^+^ cells (all progenitor cells) in the injured dorsal VZ of vehicle- and D + Q-treated aged killifish at 2 dpi. Reactive NGP proliferation is significantly augmented in D + Q-treated fish, while RG proliferation is significantly decreased. *p ≤ 0,05; Two-tailed unpaired *t*-test. Values are mean ± SEM; *n* ≥ 8 fish. **e** Number of BLBP^+^ SOX2^+^ PCNA^-^ quiescent RG, BLBP^+^ SOX2^+^ PCNA^+^ dividing RG, BLBP^-^ SOX2^+^ PCNA^-^ quiescent NGPs and BLBP^-^ SOX2^+^ PCNA^+^ dividing NGPs cells in the injured dorsal VZ of vehicle- and D + Q-treated aged killifish at 2 dpi. The total number of progenitors is significantly increased in D + Q-treated fish. This increase is mostly caused by an increased number of dividing NGPs and quiescent RGs. **p ≤ 0,01; Two-tailed unpaired *t*-test. Values are mean ± SEM; *n* ≥ 8 fish. TBI traumatic brain injury, dpi days post injury, q quiescent, RG radial glia, NGP non-glial progenitor, VZ ventricular zone, D + Q Dasatinib and Quercetin.

Aged fish were again i.p. injected three times with D + Q and received a TBI at the time of the last i.p. injection, to reduce the number of times the fish needed to be anaesthetized (Fig. 4a). Two days later, at 2 days post injury (dpi), we checked for neural progenitor cell (NPC) proliferation in the dorsal stem cell niche of the injured hemisphere (Fig. 4a), as this is the timepoint after injury when NPC proliferation peaks in young fish but not in old fish^17^. There are two kinds of NPCs present in the killifish telencephalon; RGs, characterized by the expression of typical astroglial marker genes (*blbp, gs, gfap, vim*) and NGPs, which are devoid of astroglial markers^12,17,63^. Therefore, immunohistochemistry for SOX2, PCNA and BLBP (Fig. 4b, c) allows separating the dividing RGs (SOX2^+^ PCNA^+^ BLBP^+^) from the dividing NGPs (SOX2^+^ PCNA^+^ BLBP^-^). Following D + Q treatment, aged-injured killifish showed an increased percentage of dividing NGPs, and a decreased percentage of dividing RGs, compared to vehicle-treated controls (NGPs: 24,1% ± 2,7 versus 15,1% ± 3, *p* = 0.045; RGs: 2,9% ± 0,7 versus 7% ± 1,6, *p* = 0.034, Fig. 4d). The cellular response in aged drug-treated fish thus phenocopied the response reported for young adult killifish, in which proliferating NGPs are significantly more present^17^. By counting the number of quiescent NGPs (SOX2^+^ PCNA^-^ BLBP^-^) we could exclude that this effect was due to loss of quiescence in NGPs; the number of quiescent NGPs was unchanged between D + Q - and vehicle-treated fish (43,4 ± 11,9 versus 37,3 ± 10,9, *p* = n.s.; Fig. 4e). Yet, the total number of progenitors present in the injured dorsal VZ of D + Q-treated fish was augmented as compared to vehicle-treated fish (376 ± 22,3 versus 272,5 ± 12,5, *p* = 0.0014; Fig. 4e). D + Q-treated fish thus increased the production of dividing NGPs.

**Fig. 5:**
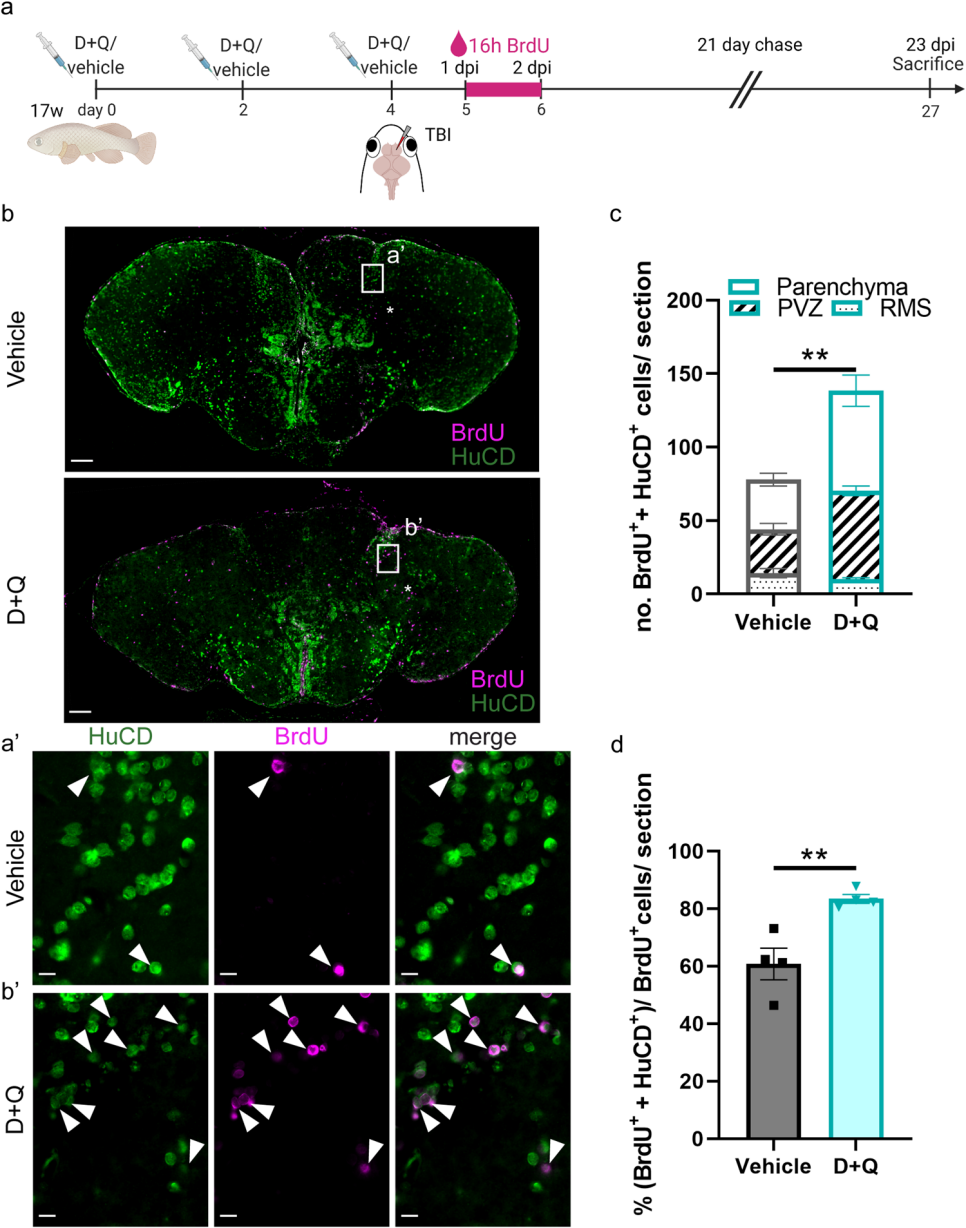
Short-term treatment of the aged killifish with D **+** Q enhances neuroregeneration after TBI. **a** Experimental setup: 17-week-old aged fish were vehicle-treated or treated with D + Q via three consecutive i.p. injections, each separated by 2 days. Together with the last injection, fish received a TBI. From 1 to 2 dpi: D + Q and vehicle fish were placed in BrdU water for 16 h: BrdU will be incorporated in the DNA of dividing cells and passed on to the progeny upon each cell division. 23 dpi: all brain samples are collected. **b** Double staining for BrdU (magenta) and HuCD (green) on coronal sections of the telencephalon of vehicle- and D + Q-treated aged fish. Scale bar: 100 µm. **a'**, **b'** Magnification of the boxed areas in (**b**). White arrowheads depict double-positive BrdU^+^ HuCD^+^ newborn neurons. Scale bars: 10 µm. **c** Number of BrdU^+^ HuCD^+^ newborn neurons in the parenchyma, PVZ of the injured dorsal pallium and near the RMS. D + Q-treated fish have significantly more newborn neurons compared to vehicle-treated fish. **p ≤ 0,01; Two-tailed unpaired *t*-test. Values are mean ± SEM; *n* = 4 fish. **d** Proportion of newborn neurons (BrdU^+^ HuCD^+^) among all BrdU^+^ cells. Of all newborn cells, ~80% are of neuron identity in D + Q-treated fish, while this is only ~60% in vehicle-treated fish. **p ≤ 0,01; Two-tailed unpaired *t*-test. Values are mean ± SEM; *n* = 4 fish. TBI traumatic brain injury, i.p. intraperitoneal, D + Q Dasatinib and Quercetin, PVZ periventricular zone, RMS rostral migratory stream, dpi days post injury.

With NGPs being responsible for the production of new neurons^12,17,63^, we next examined if more new neurons were generated and if these neurons could migrate to the injury site. We treated aged killifish with the same D + Q and TBI regimen (Fig. 5a). Next, we placed D + Q- and vehicle-treated killifish in BrdU water for 16 h in between 1 and 2 dpi, when reactive proliferation is most intense^17^. BrdU will be incorporated in the DNA of all cells passing through the S-phase and is passed on to their progeny (Fig. 5a). After a 21-day chase period, we identified the neuronal progeny in the injured hemisphere via BrdU and HuCD double immunolabelling (Fig. 5b). The total number of mature neurons born in the first days post injury (BrdU^+^ HuCD^+^) was significantly higher in D + Q-treated fish compared to vehicle fish (138,4 ± 13,4 versus 77,9 ± 6,5, *p* = 0.007, Fig. 5c). The same was true for neurons counted in the periventricular zone (PVZ; 60,2 ± 3,2 versus 29,9 ± 3,9, *p* = 0.001), as well as in the parenchyma of the injured hemisphere (68,1 ± 10,6 versus 33,8 ± 4,4, *p* = 0.02, Fig. 5c). Hence, not only more neurons were born upon D + Q administration, but more neurons were able to also reach the injury site in the parenchyma and to mature to establish restorative growth. Also here, D + Q-treated aged killifish phenocopied the regeneration response of untreated young killifish, for which we reported a higher number of NGP-derived newborn neurons that migrated and differentiated in the parenchyma in comparison to aged killifish^17^. In addition, the proportion of newborn cells (all BrdU^+^ cells) that adopt neuron identity (HuCD^+^) was significantly higher in D + Q-treated fish compared to vehicle fish (83.4% ± 3.1 versus 60.8% ± 11, *p* = 0.007, Fig. 5d). These results might relate to more newborn neurons reaching a mature state in D + Q-treated fish.

All in all, our results show that a short D + Q treatment revives the stem cell niches, re-potentiating the generation of proliferating NGPs and the production and migration of newly maturing neurons in an already aged brain context.

### D **+** Q treatment alters morphology of microglia, but does not prevent glial scarring of the aged telencephalon after TBI

**Fig. 6:**
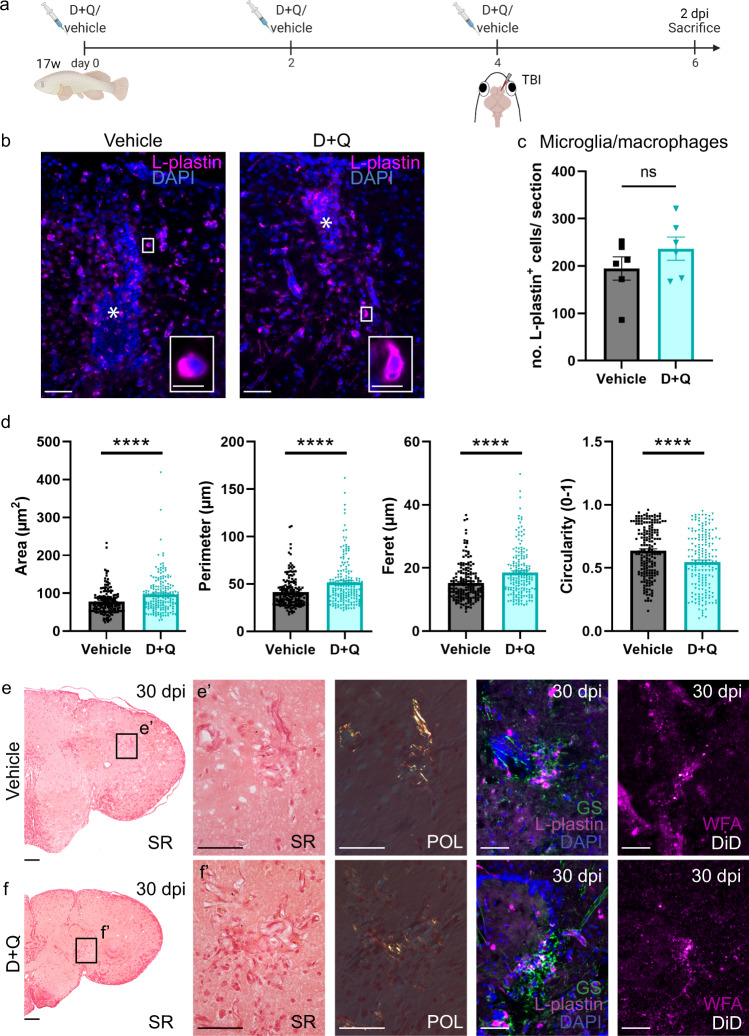
D **+** Q treatment alters microglia morphology, but does not prevent glial scarring after TBI. **a** Experimental setup: 17-week-old aged fish were vehicle-treated or treated with D + Q via 3 consecutive i.p. injections, each separated by 2 days. Together with the last injection, fish received a TBI. Samples were made 2 days after the last injection+TBI (at 2 dpi). **b** Staining for L-plastin (magenta) with DAPI (blue) on coronal brain sections of vehicle- and D + Q-treated aged killifish at 2 dpi. The injury site is marked by an asterisk. Higher magnification of individual L-plastin^+^ microglia/macrophages are presented in the right bottom corner. Scale bars: in **b**: 50 µm; in boxed areas: 10 µm. **c** Absolute number of L-plastin^+^ microglia/macrophages in the injured hemisphere of vehicle- and D + Q-treated aged killifish at 2 dpi. Both conditions show a similar number of microglia/macrophages early after brain injury. Two-tailed unpaired *t*-test. Values are mean ± SEM; *n* = 6 fish. **d** Measurement of the area (µm^2^), perimeter (µm), feret (µm) and circularity of individual microglia/macrophages in vehicle- versus D + Q-treated aged fish at 2 dpi. Microglia/macrophages in the D + Q-treated fish are larger and have more distance between their processes, suggesting a ramified morphology in contrast to microglia/macrophages in the vehicle condition, which are more circular, suggesting an ameboid morphology. ****p ≤ 0.0001; Two-tailed Mann–Whitney test. Values are mean ± SEM; *n* = 180 microglia/macrophages. **e**, **f** Glial scarring is still present in the injured hemisphere of vehicle-treated (**e**) and D + Q-treated (**f**) aged killifish at 30 dpi. Picro sirius red (SR) staining and WFA staining are used to respectively visualize collagen and proteoglycan deposition at the injury site (*n* ≥ 3 fish). Only when the SR staining is exposed to polarized light (POL), the natural birefringence of the collagen is enhanced and collagen fibers can be visualized as red/yellow (collagen type I) or green color (collagen type III)^83^. Double staining for L-plastin (red) and GS (green) with DAPI (blue) is used to visualize the glial component of the glial scar; a cluster of L-plastin^+^ microglia/macrophages in the center, surrounded by GS^+^ RG fibers (*n* ≥ 3 fish). Scale bars in SR overview pictures: 100 µm; in insets: 50 µm. i.p. intraperitoneal, D + Q Dasatinib and Quercetin, TBI traumatic brain injury, dpi days post injury, SR Picro sirius red, POL polarized light, WFA Wisteria Floribunda agglutinin.

In the context of normal aging, losing the ability to recover from injury via neuroregeneration in the aged killifish telencephalon goes hand in hand with a local and high inflammatory reaction and glial scarring^17^. By immunostaining for L-plastin, a marker for microglia/macrophages, we could count the number of microglia/macrophages at 2 dpi (Fig. 6a), a timepoint with ~33% more L-plastin^+^ microglia/macrophages surrounding the injury site in aged fish compared to young adult fish^17^. We observed a similar accumulation of microglia/macrophages in D + Q- versus vehicle-treated aged killifish (Fig. 6b, c), as well as a similar amount of dividing microglia/macrophages (Supplementary Fig. 2). Morphometric analyses revealed that microglia/macrophages in the D + Q-treated fish obtained a larger area, perimeter and feret diameter (Fig. 6d, Supplementary Fig. 3), indicative of a more complex and ramified morphology. Microglia/macrophages in the vehicle-treated fish in contrast, had a more circular morphology, thus displaying a more ameboid shape (Fig. 6d).

In conclusion, short-term D + Q treatment of the aged telencephalon does not influence microglia/macrophage numbers near the injury site, but rather the morphology of these immune cells after injury.

At 30 dpi, we probed for the presence of a glial scar by Picro sirius red staining and WFA staining to visualize collagen fibers and proteoglycan deposition respectively. We also stained for L-plastin together with GS (Fig. 6e, f), to visualize the glial component of the scar^17^. In young adult killifish, there is no formation of a glial scar after TBI. Contrary to our expectations a glial scar was formed in the D + Q-treated aged killifish. It seems that a short treatment regimen with D + Q thus specifically influences the neurogenic output of the NGPs and changes the inflammatory response after injury, without preventing scarring of the tissue.

## Discussion

The African turquoise killifish is a unique biogerontology model, as it combines a very short lifespan with a vertebrate physiology while expressing the cellular and molecular hallmarks of human aging, including an impaired neuroregenerative potential with advancing age^1–3,14–19^. This makes the killifish a competitive vertebrate preclinical model, in which new anti-aging strategies can be tested within a reasonable timeframe. Here, we have exploited the killifish to find a new strategy that could increase the aged brain’s resilience to brain injuries or neurodegeneration. Using a proteomics approach, we identified increased cellular senescence as a hallmark of the aged brain. We revealed a significant senescent cell burden in the aged telencephalon, which can be reduced by a short treatment with the senolytic drug combination D + Q, even when only starting at very old age. The treatment increased the neurogenic power and altered the inflammatory reaction of the aged brain, holding great potential toward better protection against TBI. We predict these results will fuel the establishment of new therapies where senolytics can be used to improve neurorepair of the aged injured or diseased brain.

Differential proteomics is a reliable way to detect changes in protein levels. Transcript and protein levels get decoupled with aging^13^, meaning that a change in transcript level does not always result in a change of the corresponding protein level and vice versa. Detecting changes in protein levels can therefore be more interesting as it avoids overestimating physiologically relevant changes, as well as events remaining undetected. The proteomics dataset acquired in the current study showed typical, but also novel, age-related differences in protein levels. The increased levels of the glial intermediate filament proteins GFAP and VIML for instance, is highly associated with the aging brain^15,39,40^. Of interest, the aged killifish telencephalon showed higher levels of SNCA, which might point to possible synucleinopathies. SNCA is a major component of the Lewy bodies found in the brain of Parkinson’s disease (PD) patients^41^ and SNCA levels are elevated in prefrontal cortex samples of PD patients^42^. The killifish thus might develop a spontaneous Parkinson disease-like phenotype with aging. Age-dependent degeneration of noradrenergic neurons and possibly dopaminergic neurons has been reported in the killifish^18,19^, together with the presence of SNCA inclusion bodies^18^.

Pathway analysis of the differential proteins identified enriched pathways including *ribosome*, *spliceosome*, *base excision repair*, *Insulin and mTOR signaling* pathways and *cellular senescence*. Many of these pathways were also observed in the dataset of Sacramento and colleagues (2020) that compared protein levels between 5-week-old (young) and 12-week-old (middle-aged) killifish, as well as between 12-week-old and 39-week-old (aged) killifish^13^. The occurrence of cellular senescence was however not detected in this study, which might be attributed to the use of a different, long-lived, killifish strain, the comparison of different ages or a different pathway analysis method. Evaluation of the relative expression of typical senescence markers and the activity of SA-βgal did confirm our proteomics data. While the young telencephalon was almost devoid of senescent cells, the aged telencephalon contained large numbers of senescent cells. A significant number of these cells was found at the ventricular surface, which is the stem cell niche of the telencephalon^15,63^. As such, NGPs and RGs were most likely among the cell types becoming senescent as these progenitor types occupy the VZ^15,63^. NGPs are essential for neuroregeneration since they are responsible for the production of new neurons in the killifish telencephalon^17,63^. In addition, while most killifish RGs are quiescent, a small subpopulation of RGs retains some minor proliferative capacity, presumably to generate new NGPs^12,17,63^. Having both these populations in an arrested, senescent state most likely disturbs stem cell niche homeostasis and hampers neuroregeneration. Only recently, Fatt and colleagues (2022) also reported on the accumulation of senescent cells, of especially the progenitor type, in the aged mouse dentate gyrus^64^. This accumulation coincided with reduced neurogenesis and cognitive ability^64^. They also speculate that the large proportion of senescent progenitors restricts neurogenesis by, on the one hand, being unable to enter the cell cycle, and on the other hand, by influencing their non-senescent neighboring cells through a pro-inflammatory SASP^65^.

Using the senolytic drug combination D + Q, we observed an increase in apoptotic cell number, which correlated to a decreased expression of a set of senescence markers, suggesting D + Q specifically kill senescent cells, as already shown by several other studies^47,53–60^. Using a short treatment of 1 week and starting at very old age, we could show that D + Q can enhance the regenerative ability of aged fish, reminiscent of what normally takes place in young killifish. Remarkably, we discovered that D + Q treatment resulted in the increased production of new proliferating NGPs, rather than driving existing NGPs to reenter the cell cycle. The increase in proliferating NGPs was mirrored by a decline in proliferating RGs, which might relate to RGs giving rise to new NGPs by symmetric or asymmetric division early after injury. The possibility that RGs act as quiescent progenitors, generating NGPs in the killifish brain, has been postulated before^12,17,63^, yet mechanistic evidence is still lacking. NGPs also have a lot of self-renewal potential and thus might also be responsible for the increase in dividing NGPs^17,63^.

The activation of the pallial progenitors could be directly caused by the pro-proliferative effect of quercetin on stem cells^66–68^. Alternatively, the activation could be indirectly caused by D + Q through the removal of neighboring senescent cells, the SASP or by altering injury-induced inflammation. Indeed, in D + Q-treated aged fish, microglia obtained a more elongated morphology at 2 dpi, indicating a more resting state. D + Q potentially shifted the inflammatory response from a prolonged to an acute one, which is highly linked to the activation of stem cells and a pro-regenerative environment^69,70^. In contrast to the chronic reaction in mammals, the inflammatory response is indeed acute in regeneration-competent vertebrates like zebrafish^69,70^ and young adult killifish^17^. When this acute reaction is altered via suppressing drugs^69^ or granulin-deficiency in zebrafish^71^, or due to aging in killifish^17^, neuroregenesis is severely impeded.

The D + Q induced rise in dividing NGPs resulted in more newborn neurons that could migrate to the injury site in D + Q-treated aged fish. These new neurons were positive for HuCD, suggesting that they also differentiated into a mature state. The question if these newly matured neurons functionally integrate in the existing circuitry remains unanswered. Electrophysiology or calcium imaging assays are not yet optimized in killifish. Still, we predict that future endeavors will discern that D + Q can induce functional integration of the newborn neurons in the aged injured brain.

Glial scarring occurred independent of D + Q treatment in the aged fish. A possible explanation for this observation might be the involvement of injury-induced senescent cells in glial scarring. In regeneration-competent vertebrates, like adult zebrafish and salamanders, injury-induced senescent cells appear at the injury site but are quickly removed by macrophages^72–74^. In regeneration-incompetent vertebrates on the contrary, injury-induced senescent cells are persistent^74^. The removal of these persistent senescent cells with senolytics from 5 to 14 days after spinal cord injury boosted neurorepair. The treated mice showed axon preservation, reduced inflammation near the injury site, reduced fibrotic scarring and improved functional outcome^74^. In our study, D + Q was never administered during the regeneration process and probably did not target the potential injury-induced senescent cells. Our study hence decouples autonomous-senescence from injury-induced senescence and shows that eliminating autonomous-senescence can boost neurogenic output in the injured aged brain, despite glial scarring.

In conclusion, we have demonstrated the validity of the killifish as a preclinical model to elucidate and reverse the restrictions on neuroregeneration caused by aging. Furthermore, we have proven that even very short treatments with D + Q, at a time when autonomous cellular senescence has already kicked-in, can increase the neurogenic potential, opening up the opportunity to treat brain injury and neurodegenerative disease by enhancing the regenerative power of the aged brain.

## Methods

### Fish strain and housing

All experiments were performed on young adult and aged (6-week- and 17 till 18-week-old) female African turquoise killifish (Nothobranchius furzeri, strain GRZ-AD)^17^. These ages were based on a previously published survival curve^17^, representing 100% and 76% survival respectively. The median lifespan is reached at 24 weeks of age^17^. Fish were raised in a ZebTEC Multi-Linking Housing System (Tecniplast) as described before^17^. All experiments were approved by the KU Leuven ethical committee in accordance with the European Communities Council Directive of 22 September 2010 (2010/63/EU) and the Belgian legislation (KB of 29 May 2013).

### Proteomic analysis of young and aged telencephalon samples

#### Protein sample preparation

Young adult and aged fish were euthanized in 0.1% buffered tricaine (MS-222, Sigma-Aldrich, diluted in system water). Brains were extracted and the right hemisphere of the telencephalon was quickly dissected and placed on dry ice. Samples were immediately stored at −80 °C. Next, samples were homogenized using drill-driven, sterile, disposable pestiles (Argos technologies) in 500 µL RIPA buffer (150 mM NaCl, 50 mM Tris, 1% Triton, 0.5% Na deoxycholate, 0.1% SDS, 4% protease inhibitor, pH 8). The samples were shaken for 2 h at 4 °C before being centrifuged for 20 min at 12,000 g and at 4 °C. Hereafter, the supernatant was stored at −80 °C and shipped to the Centre for proteomics, Prof. Dr. G. Baggerman research group (University of Antwerp). The protein concentration was defined using the Pierce™ BCA Protein Assay Kit. 20 µg of each sample was reduced at 4 °C for 30 min by addition of 10 mM TCEP. Then, a modified FASP procedure was used for detergent removal and digestion. Briefly, samples were mixed with 8 M urea in Tris-HCl and loaded on a 30 kDa filter (Abcam) and centrifuged at 14,000 g for 10 min. The proteins on the filter were washed by addition of urea solution on the filter followed by centrifugation at 14,000 g for 10 min. Proteins were alkylated by addition of 50 mM iodoacetamide in urea solution and incubated for 30 min at RT in the dark. Then, the spin filter was again washed three times with ureum solution, followed by three washes with a 50 mM ammonium bicarbonate solution prior to digestion with two consecutive enzymatic digestions. The first digestion was performed by addition of LysC in ammonium bicarbonate (ratio LysC:protein 1:40) during 2 h at 30 °C. After collection of the digest by centrifugation at 14,000 g for 10 min, the second digestion was performed ON at 37 °C with trypsin (ratio trypsin:protein 1:100). The digest was again collected by centrifugation at 14,000 g for 10 min followed by addition of 50 mM ammonium bicarbonate solution to the filter which was again collected by centrifugation at 14,000 g for 10 min. Finally, 50 µL of 0.5 M NaCl solution was added to the spin filter and centrifuged at 14,000 g for 10 min. The four collected filtrate fractions were pooled and after acidification with formic acid desalted on Pierce™ C18 Spin Columns (Thermo Scientific). After which samples were dried in a vacuum concentrator and reconstituted in 20 µL of 1% Acetonitrile, 0.1% formic acid. The concentration of the peptides was determined by the Pierce™ Quantitative Colorimetric Peptide Assay.

#### Reverse phase liquid chromatography and mass spectrometry

The equivalent of 1 µg of peptides dissolved in 10 µl of 1% ACN and 0.1% FA were separated on a Nano Acquity Ultra Performance LC system (Waters) fitted with a µPAC trapping column (Pharmafluidics) and a µPAC 200 cm column (Pharmafluidics). The sample was loaded onto the trap column in 3 min at 10 µl/min in 99% solvent A, 1% solvent B (solvent A is 1% ACN, 0.1% FA in UPLC/MS grade water (Biosolve), solvent B is 0.1% FA in 99% ACN). The flow over the main column was 750 nL/min and the column was heated to 50 °C. After an isocratic flow of 4 min at 1% B, the concentration of B increased in 80 min to 40% B then to 100% B in 5 min. After again an isocratic flow of 5 min at 100% B, the concentration of B was decreased in 3 min to 1% which was followed by 34 min of equilibration at 1%. The nano-LC was coupled online with the mass spectrometer using a stainless-steel nano-bore Emitter (Thermo scientific) coupled to a Nanospray Flex ion source (Thermo Scientific). The Q Exactive Plus (Thermo Scientific) was used in data dependent analysis (DDA) method. The standard shotgun method was set up in MS/MS mode where a full scan spectrum (350–1850 m/z, resolution 70,000) was followed by a maximum of twenty HCD tandem mass spectra in the orbitrap, at a resolution of 17,500. A maximum inject time of 100 ms was set in the full MS, and 80 ms in MS2. The normalized collision energy used was 28 and the minimal AGC target was set at 1.7 × 103. An isolation window of 1.6 m/z and isolation offset of 0.3 m/z was applied. We assigned a dynamic exclusion list of 20 s.

#### Proteomics data analysis

Identification of proteins was performed with MaxQuant^75^ by searching against the Uniprot *Nothobranchius furzeri* database with the following parameters. Trypsin was indicated as digestive enzyme with a maximum of two missed cleavages. Fixed modification was set to cysteine carbomidomethylation, and variable modifications to methionine oxidation and N-protein acetylation. The FDR for PSM and protein identification was 1%. The analysis was specified to label-free quantification (LFQ) with a minimal ratio count of 2 for unique or razor peptides. Raw output data from MaxQuant was further analyzed using Perseus v.1.6.15^76^. Protein identifications only identified by a single modified peptide, as well as decoys and possible contaminants were removed from the data frame. Outliers were removed from the dataset based on PCA (Supplementary Fig. 4a, b). LFQ-values were log2 transformed and annotated per condition: young and aged. Proteins with a minimal of 70% valid values in total were statistically analyzed. A volcano plot-based double sided *t*-test was performed with FDR of 0,05. We identified 1413 proteins, of which 398 had significant different levels between young and aged brain samples (Supplementary Table 1). These DPs were divided into increased or decreased DPs and both lists were entered into STRING^43^ for pathway enrichment analysis (Supplementary Table 2 and 3) using *Danio rerio* as background species.

### Dasatinib and quercetin (D + Q) administration

Fish were anesthetized in 0,03% buffered tricaine (MS-222, Sigma-Aldrich) and positioned in a cold moist sponge with their belly facing upwards. D and Q are both water-insoluble drugs. As such, D + Q was not administered via the tank water, but via i.p. injection. We did not choose for D + Q administration via oral gavage because this is very time-consuming, labor intensive and causes stress in the animals. A stock solution was first made in 100% DMSO, which was diluted 100 times in saline (0,9% NaCl) to obtain a working solution. Using a custom Hamilton syringe with 33-Gauge needle, 10 µL of D + Q working solution was i.p. injected. The working solution consists of 0,1 mg/mL D and 1 mg/mL Q in 1% DMSO. The concentration of D + Q was calculated based on the body weight of the fish. In rodents, 5 mg/kg D and 50 mg/kg Q is often administered via oral gavage^77–79^. We observed that this amount of drug per body weight was detrimental for the fish. We therefore lowered the amount 10 times (0.5 mg/kg D and 5 mg/kg Q), which resulted in the expected senescence reduction of 30% without affecting fish health. Vehicle-treated killifish were i.p. injected with 1% DMSO (diluted in 0,9% NaCl). The solutions were always vigorously vortexed before use. After the injection, fish were placed back into their home tanks and water was stowed over the gills to facilitate recovery.

### Stab-wound injury

Fish were anesthetized in 0,03% buffered tricaine (MS-222, Sigma-Aldrich) and positioned in a cold moist sponge. A very small opening was created above the right hemisphere of the telencephalon in between the eyes. A custom Hamilton 33-Gauge needle was first dipped into Vybrant DiD cell-labeling solution (Thermo Fisher, V22887) to mark the injury site on sections, before being pushed through the skull into the dorsal pallium of the right hemisphere of the telencephalon. For recovery, fish were placed back into their home tanks and water was stowed over the gills^17^.

### BrdU labeling

Fish were submerged into 5-Bromo-2’-deoxyuridine (BrdU, Sigma-Aldrich, B5002) water at a concentration of 7,5 mM to label dividing cells and their progeny. After 16 h of BrdU labeling, fish were placed back into their home tanks for 21 days.

### Tissue fixation and processing

Fish were euthanized in 0,1% buffered tricaine and perfused with PBS and paraformaldehyde (4% PFA, Sigma-Aldrich, 8.18715, in PBS)^80^. Brains were surgically removed and post-fixed for 12 h in 4% PFA at 4 °C. Brains were washed and embedded in 30% sucrose, 1,25% agarose in PBS. With a CM3050s cryostat (Leica) 10 µm coronal sections were made. Sections were stored at −20 °C until further use.

### Picro Sirius red and SA β-gal staining

Cryostat sections were dried for 30 minutes at 37 °C to improve adhesion to the glass slides and washed in *aqua destillata* (AD). Picro sirius red staining. Cryostat sections were submerged in 70% ethanol, AD and Picro Sirius Red solution (0,1% Direct Red 80 (365548, Sigma-Aldrich) in a saturated aqueous solution of picric acid (P6477-1GA, Sigma-Aldrich). Next, sections were destained in 0,5% Acetic Acid and dehydrated in ethanol and xylol. Sections were covered with DePeX and a cover slip, and dried overnight. SA β-gal assay. Sections were washed in PBS pH 6 and incubated in SA β-gal solution overnight at 37 °C. SA β-gal staining buffer (5 mM K_3_Fe(CN)_6_, 5 mM K_4_Fe(CN)_6_.dihydrate, 3 mM MgCl_2_ in PBS pH 6) was added to GAL-X (A1007, BioChemica, 1/20 in DMF) while vortexing to create the SA β-gal solution. The following day, sections were washed in PBS pH 6 and covered with mowiol and cover slip.

### Immunohistochemistry (IHC)

All immunohistochemical stainings were performed as described before^17^. Primary antibodies used in this study are rabbit anti-L-plastin (1/500, Sigma-Aldrich, SAB2701743), rabbit anti-SOX2 (1/1000, Sigma-Aldrich, SAB2701800), mouse anti-HuC/D (1/200, Thermo Fisher, A-21271), mouse anti-PCNA (1/500, Abcam, ab29), Goat anti-BLBP (1/1000, Abcam, ab110099), rat anti-BrdU (1/1000, Abcam, ab6326), and mouse anti-GS (1/1000, Abcam, ab64613). We performed anti-WFA staining with a biotinylated Lectin from Wisteria Floribunda (WFA, 1:500, L1516, Sigma-Aldrich). TUNEL labelling was performed according to the manufacturer guidelines (Sigma-Aldrich, 11684795910).

### Microscopy

With a confocal microscope (FV1000, Olympus) all sections were searched for DiD positivity to find the injury site. A Zeiss (‘Axio Imager Z1’) fluorescence microscope equipped with a AxioCam MR R3 camera (fluorescence) was used to photograph 3 sections per animal. 20X tile scans, 20X photographs and 63X photographs were taken with the ZEN software (ZEN Pro 2012, Carl Zeiss). SA-β gal staining and collagen fibers after Picro sirius red staining were visualized with brightfield and polarized light respectively via a Leica DM6 microscope and LAS X software (Leica Microsystems). 20X tile scans and 63X photographs were taken. For publication, channels were equally intensified for all conditions using Adobe Photoshop SC5 (Adobe Systems).

### Real-time quantitative polymerase chain reaction (RT qPCR)

Young and aged killifish were euthanized in 0,1% buffered tricaine and the telencephalon was extracted and quickly snap-frozen on dry ice. The RNeasy Lipid Tissue Mini Kit (74804, Qiagen) was used to extract RNA, following the Manusfacturer’s instructions. cDNA was generated using oligo dT primers and Superscript III reverse transcriptase (Invitrogen). RT qPCR was performed using SYBR Green master mix (BioRad) and the CFX96/C1000 Touch Real-Time detection system (BioRad) with an annealing temperature of 60 °C. Plate set up was performed in CFX Maestro (BioRad v2.3). Reactions were run in duplo. Primer sequences for genes of interest and housekeeping genes (Supplementary Table 4) were designed based on the *N. furzeri* Transcriptome browser (www.NFIN.com). Housekeeping genes (Supplementary Table 4) were used to normalize the gene expression values. Values are quantified using the comparative Ct method, with the mean of the young/vehicle-treated cycle threshold values as the control.

### Quantification and statistical analysis

Immunopositive or SA-βgal positive cells were counted with the cell counter plugin in Image J (Fiji). OD measurements were also performed in Image J (Fiji) and divided by the OD value of the background staining (region within the section without signal). For all quantifications, an average of 3 sections was taken per animal for statistical analysis using GraphPad Prism (version 9). For injury experiments, all quantifications were performed in the injured hemisphere.

Morphology of microglia/macrophages was determined according to the method of Mitchell et al. (2018)^81^. In short, individual microglia/macrophages were outlined and measured in ImageJ (Fiji). Per condition, 180 microglia/macrophages among 6 fish were measured (on three sections per fish containing the injury site). All measurements were performed in the injured hemisphere. Four morphological parameters were analyzed; area: the area of the microglia in square microns, perimeter: the total length of the circumference of a cell, feret: the largest distance between two points along the perimeter that can be measured, circularity: a circularity closer to one illustrates a perfect circle.

Data were first tested for Gaussian normality. If assumptions were met, we used a parametric two-tailed unpaired *t*-test to compare young with aged or vehicle-treated with D + Q-treated fish. If assumptions were not met, we used the non-parametric Mann Whitney test (two-tailed). n represents the number of animals in each condition, except for the microglia morphological measurements, where n represents the number of microglia. All values are mean ± standard error of the mean (SEM). Means were statistically significantly different when *p* ≤ 0,05. See supplementary Table 5 for all information on the statistical data.

### Reporting summary

Further information on research design is available in the Nature Research Reporting Summary linked to this article.

## Supplementary information

Supplementary information

Reporting Summary

Supplementary Table 1

Supplementary Table 2

Supplementary Table 3

## Data Availability

Further information and requests for resources, such as killifish, should be directed to the Lead Contact, Lutgarde Arckens (lut.arckens@kuleuven.be). The mass spectrometry proteomics data have been deposited to the ProteomeXchange Consortium (http://proteomecentral.proteomexchange.org) via the PRIDE partner repository^82^ with the dataset identifier PXD609575 and 10.6019/PXD036437.
