# Supplementary material for: A short dasatinib and quercetin treatment is sufficient to reinstate potent adult neuroregenesis in the aged killifish
Source: NPJ Regen Med. 2023 Jun 16;8:31. doi: 10.1038/s41536-023-00304-4 (PMC10275874; doi:10.1038/s41536-023-00304-4)
Supplement: Supplementary file 1 — Supplementary information [file 41536_2023_304_MOESM1_ESM.pdf]

Supplementary figures

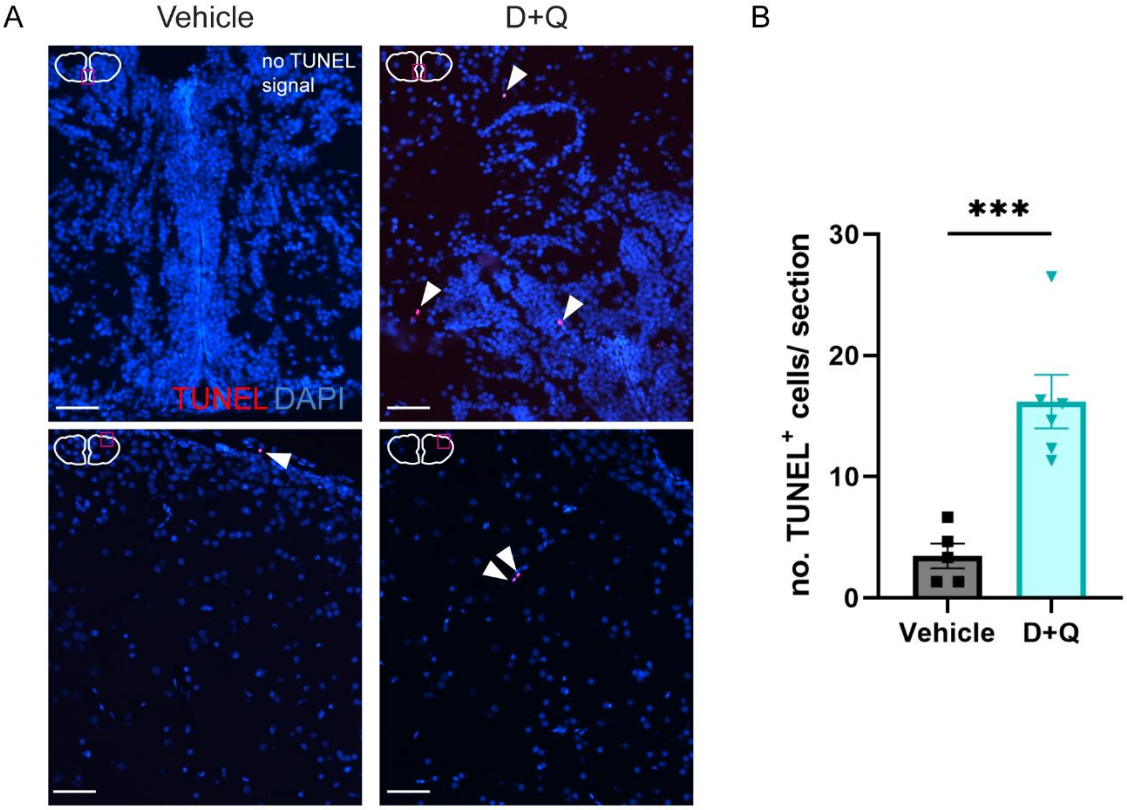

Supplementary figure 1: Apoptosis is increased six hours after D+Q treatment. (A) Staining for TUNEL (red) with DAPI (blue) on coronal brain sections of aged killifish six hours after vehicle or D+Q administration. An increased number of TUNEL$^+$ apoptotic cells is detected in D+Q-treated fish six hours after administration, which matches with the bioavailability of the two drugs (both have half-life elimination times <12 hours). Scale bars: 50 µm. The region where the image (20x) was taken on the section is depicted in the left top corner of each panel. (B) Absolute number of TUNEL$^+$ apoptotic cells in vehicle- and D+Q-treated aged killifish six hours after administration. The number of TUNEL$^+$ apoptotic cells is four times higher in D+Q-treated fish compared to vehicle-treated fish. ***p≤0,001; Two-tailed unpaired t-test. Values are mean ± SEM; n≥5 fish. D+Q: Dasatinib and Quercetin.

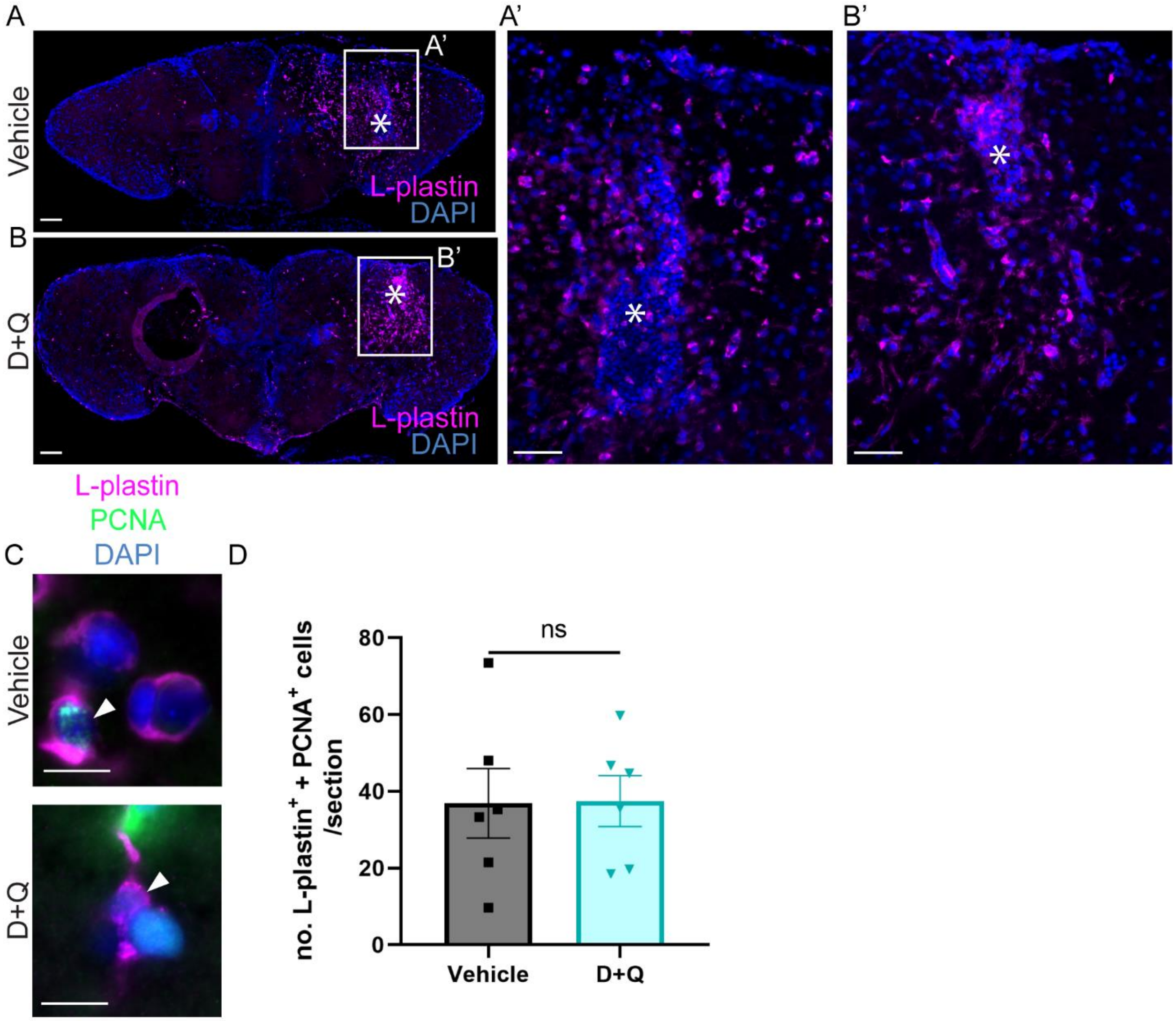

Supplementary figure 2: D+Q treatment does not influence proliferation of microglia after injury. Related to Figure 6. (A,B) Staining for L-plastin (magenta) with DAPI (blue) on coronal brain sections of vehicle- (A) and D+Q-treated (B) aged killifish at 2 dpi. The injury site is marked by an asterix. Scale bars: 100 µm. (A', B') Magnification of the boxed areas in (A) and (B) respectively. Scale bars: 50 µm. (C) Staining for L-plastin (magenta) and PCNA (green) with DAPI (blue) on coronal brain sections of vehicle- and D+Q-treated aged killifish at 2 dpi. A portion of L-plastin$^+$ microglia are PCNA$^+$ and thus dividing. Scale bars: 10 µm. (D) Absolute number of double L-plastin$^+$ PCNA$^+$ dividing microglia/macrophages in vehicle- and D+Q-treated aged killifish at 2 dpi. Both conditions show a similar number of dividing microglia early after brain injury. Two-tailed unpaired t-test. Values are mean ± SEM; n≥5 fish. D+Q: Dasatinib and Quercetin.

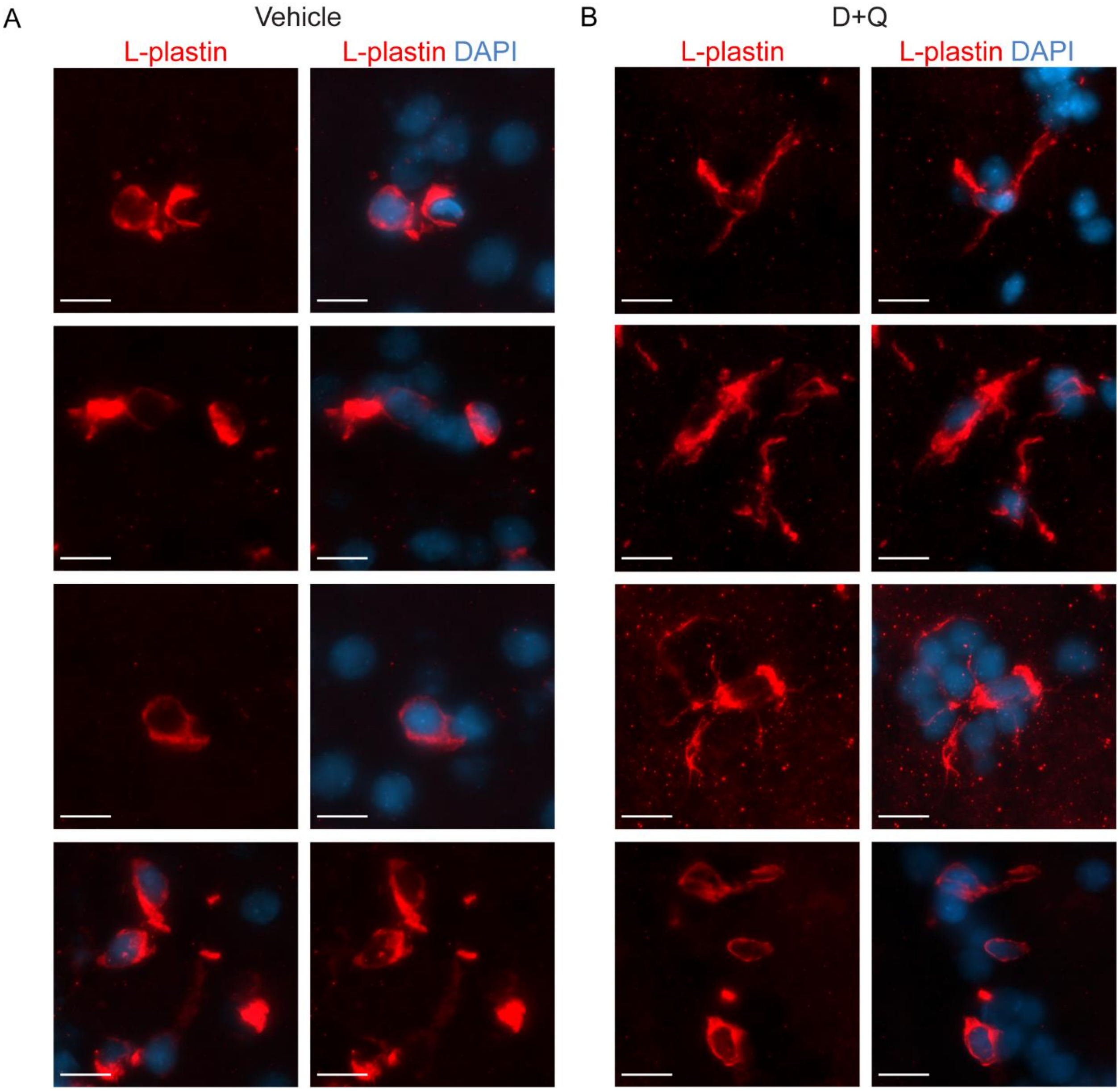

Supplementary figure 3: Examples of microglia/macrophages with different morphology in vehicle- and D+Q- treated fish. Related to Figure 6. (A,B) Staining for L-plastin (red) with DAPI (blue) on coronal brain sections of vehicle- (A) and D+Q-treated (B) aged killifish at 2 dpi. Scale bars: 10 µm. D+Q: Dasatinib and Quercetin.

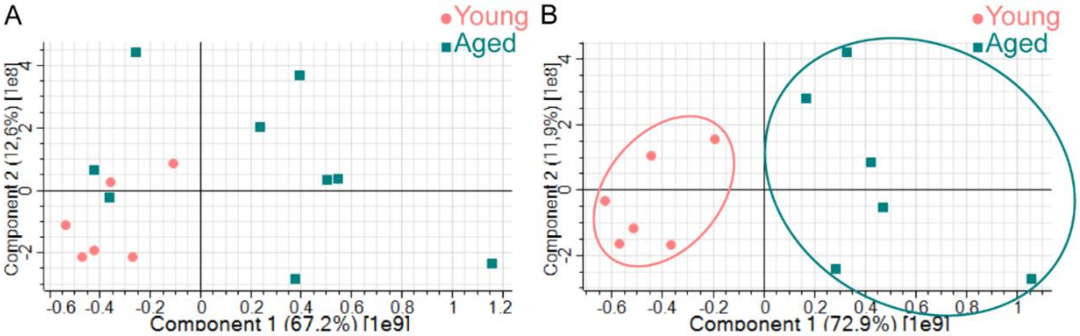

Supplementary figure 4: Principal component analysis. Related to the Methods section. (A) Principal component analysis (PCA) reveals a greater variation between aged samples in comparison to young samples. (B) Based on PC2, we removed three outliers from the aged dataset, which resulted in an equal sample size for young and aged fish (n=6) for proteomic analysis.

Supplementary tables

Supplementary table 1: LFQ of aged versus young telencephalon samples of the killifish. Statistically differential levels of proteins are shown in column A (n=6 fish).

Provided online as an .xlsx file.

Supplementary table 2: KEGG Pathways related to lower protein levels.

Provided online as an .xlsx file.

Supplementary table 3: KEGG Pathways related to higher protein levels.

Provided online as an .xlsx file.

Supplementary table 4: Overview of primers used in RT-qPCR experiments.

Gene Name	FWD primer (5'-3')	REV primer (5'-3')
p27	CGAGTTTCAAACGGGAGTC	CCCGCATGTGTTCGTTAAA
p21	GACTGCCCTGCGTAAAGAT	CAGAGGTTTGTCGGAGAAGAA
p53	CCATCAGAACAAGGACTCATC	AGTCACACTCTGCCTCTT
mdm2	GGACGAGAGGACAGAAGAA	AATCACACACCACGACAAG
il8	ACAAATCCTGACCACAAGTAG	ATCGTATTCACCATCATGTCTC
**Housekeeping genes**		
ef1a	ACTCTGGCATTGTCGTTTAG	AGTTACCAGCAGCTTTCTTC
tuba	CAGATGGTCAAGTGTGATCC	AGTTGGAGGCTGGTAGTT
tbp	CTCACAGTTACAGGACCAAAC	TCCGAACCTCAAAGAGAAGA

Supplementary table 5: Overview of the statistical data generated. MG: microglia/macrophages. All tests are two-tailed.

Fig.	*Young versus Aged*	*Passed normality*	*Test*	*p value*	*n Young*	*n Aged*	*mean Young*	*mean Aged*	*SD Young*	*SD Aged*	*SEM Young*	*SEM Aged*	*effect size*	*alpha*
2C	OD of SA B-gal	Yes	Unpaired T-test	0,0064	4	5	1,02	1,04	0,007	0,008	0,003	0,004	2,56	0,05
	number of SA B-gal	No	Mann Whitney	0,016	4	5	27,8	150,5	6,3	29,5	3,2	13,2	5,76	0,05
2D	p21 qPCR	Yes	Unpaired T-test	<0,0001	5	5	1,01	4,3	0,2	0,8	0,07	0,37	5,14	0,05
	p27 qPCR	Yes	Unpaired T-test	0,0053	5	5	1,03	1,9	0,3	0,4	0,14	0,17	2,40	0,05
	p53 qPCR	Yes	Unpaired T-test	0,0051	4	6	1,35	9,2	1,1	3,95	0,56	1,61	2,72	0,05
	mdm2 qPCR	Yes	Unpaired T-test	0,14	4	6	1,19	4,3	0,8	3,7	0,42	1,5	1,17	0,05
	il8 qPCR	Yes	Unpaired T-test	0,0349	4	5	1,02	4,9	0,2	2,9	0,11	1,3	1,87	0,05
Fig.	*Vehicle versus D+Q*	*Passed normality*	*Test*	*p value*	*n Vehicle*	*n D+Q*	*mean Vehicle*	*mean D+Q*	*SD Vehicle*	*SD D+Q*	*SEM Vehicle*	*SEM D+Q*	*effect size*	*alpha*
3D	OD of SA B-gal	Yes	Unpaired T-test	0,0397	5	6	1,05	1,04	0,01	0,01	0,006	0,005	1,65	0,05
	number of SA B-gal	Yes	Unpaired T-test	0,023	5	6	109,8	76,2	21,8	19,02	9,7	7,8	1,65	0,05
3E	p21 qPCR	No	Mann Whitney	0,0159	5	4	1,108	0,5	0,6	0,02	0,27	0,01	1,43	0,05
	p27 qPCR	Yes	Unpaired T-test	0,2206	5	5	1,19	0,67	0,9	0,18	0,38	0,08	1,43	0,05
	p53 qPCR	Yes	Unpaired T-test	0,0048	13	13	1,11	0,63	0,52	0,2	0,14	0,05	1,22	0,05
	mdm2 qPCR	No	Mann Whitney	0,001	13	12	1,14	0,49	0,58	0,3	0,16	0,09	1,40	0,05
	il8 relative qPCR	Yes	Unpaired T-test	0,3821	5	5	1,39	0,82	1,3	0,4	0,59	0,16	0,58	0,05
4D	% of dividing RG	Yes	Unpaired T-test	0,034	8	9	7	2,9	4,6	2,2	1,6	0,7	1,11	0,05
	% of dividing NGP	Yes	Unpaired T-test	0,045	8	9	15,1	24,1	8,5	8,3	3	2,7	1,13	0,05
4E	number of progenitors	Yes	Unpaired T-test	0,0014	8	9	272,50	376,00	35,40	66,99	12,50	22,30	1,93	0,05
5C	number of newborn neurons	Yes	Unpaired T-test	0,007	4	4	77,9	138,4	13,04	26,9	6,5	13,4	2,97	0,05
	RMS	Yes	Unpaired T-test	0,2521	4	4	14,3	10,2	6,2	1,8	3,1	0,9	0,93	0,05
	PVZ	Yes	Unpaired T-test	0,001	4	4	29,9	60,2	7,9	6,3	3,9	3,2	4,76	0,05
	parenchyma	Yes	Unpaired T-test	0,02	4	4	33,8	68,1	8,7	21,2	4,4	10,6	2,20	0,05
5D	% of neurons among newborn cells	Yes	Unpaired T-test	0,007	4	4	60,8	83,4	11	3,1	11	3,1	2,85	0,05
6C	number of MG	Yes	Unpaired T-test	0,2564	6	6	194,8	236,6	60,06	60,04	24,5	24,5	0,70	0,05
6D	Area MG	No	Mann Whitney	<0,0001	180	180	77,7	97,1	32,3	49,1	2,4	3,7	0,48	0,05
	Perimeter MG	No	Mann Whitney	<0,0001	180	180	41,2	51,5	15,6	24,6	1,2	1,8	0,50	0,05
	Feret MG	No	Mann Whitney	<0,0001	180	180	15,3	18,5	5,7	7,6	0,4	0,6	0,49	0,05
	Circularity MG	No	Mann Whitney	<0,0001	180	180	0,64	0,55	0,2	0,2	0,02	0,02	0,43	0,05
S2	number of dividing MG	Yes	Unpaired T-test	0,9595	6	6	36,9	37,5	22,2	16,2	9	6,6	0,05	0,05
S1	Number of TUNEL+ apoptotic cells	Yes	Unpaired T-test	0,0009	5	6	3,5	16,2	2,3	5,4	1,02	2,2	3,06	0,05
