# Supplementary material for: A short dasatinib and quercetin treatment is sufficient to reinstate potent adult neuroregenesis in the aged killifish
Source: NPJ Regen Med. 2023 Jun 16;8:31. doi: 10.1038/s41536-023-00304-4 (PMC10275874; doi:10.1038/s41536-023-00304-4)
Supplement: Supplementary file 2 — Reporting Summary [file 41536_2023_304_MOESM2_ESM.pdf]

nature portfolio

Corresponding author(s): L. Arckens

Last updated by author(s): May 3, 2023

Reporting Summary

Nature Portfolio wishes to improve the reproducibility of the work that we publish. This form provides structure for consistency and transparency in reporting. For further information on Nature Portfolio policies, see our Editorial Policies and the Editorial Policy Checklist.

Statistics

For all statistical analyses, confirm that the following items are present in the figure legend, table legend, main text, or Methods section.

n/a	Confirmed	
☐	☒	The exact sample size (*n*) for each experimental group/condition, given as a discrete number and unit of measurement
☐	☒	A statement on whether measurements were taken from distinct samples or whether the same sample was measured repeatedly
☐	☒	The statistical test(s) used AND whether they are one- or two-sided<br>*Only common tests should be described solely by name; describe more complex techniques in the Methods section.*
☒	☐	A description of all covariates tested
☐	☒	A description of any assumptions or corrections, such as tests of normality and adjustment for multiple comparisons
☐	☒	A full description of the statistical parameters including central tendency (e.g. means) or other basic estimates (e.g. regression coefficient) AND variation (e.g. standard deviation) or associated estimates of uncertainty (e.g. confidence intervals)
☐	☒	For null hypothesis testing, the test statistic (e.g. *F*, *t*, *r*) with confidence intervals, effect sizes, degrees of freedom and *P* value noted<br>*Give P values as exact values whenever suitable.*
☒	☐	For Bayesian analysis, information on the choice of priors and Markov chain Monte Carlo settings
☒	☐	For hierarchical and complex designs, identification of the appropriate level for tests and full reporting of outcomes
☒	☐	Estimates of effect sizes (e.g. Cohen's *d*, Pearson's *r*), indicating how they were calculated

Our web collection on statistics for biologists contains articles on many of the points above.

Software and code

Policy information about availability of computer code

Data collection	Proteomics: MaxQuant (v.2.3.1.0), Perseus (v.1.6.15); Microscopy: ZEN software (ZEN Pro 2012, Carl Zeiss), LAS X software (Leica Microsystems)
Data analysis	CFX Maestro (BioRad v2.3); Image J (Fiji, v.1.54c); GraphPad Prism (v.9)

For manuscripts utilizing custom algorithms or software that are central to the research but not yet described in published literature, software must be made available to editors and reviewers. We strongly encourage code deposition in a community repository (e.g. GitHub). See the Nature Portfolio guidelines for submitting code & software for further information.

Data

Policy information about availability of data

All manuscripts must include a data availability statement. This statement should provide the following information, where applicable:

- Accession codes, unique identifiers, or web links for publicly available datasets
- A description of any restrictions on data availability
- For clinical datasets or third party data, please ensure that the statement adheres to our policy

Further information and requests for resources, such as killifish, should be directed to the Lead Contact, Lutgarde Arckens (lut.arckens@kuleuven.be). The mass spectrometry proteomics data have been deposited to the ProteomeXchange Consortium (http://proteomecentral.proteomexchange.org) via the PRIDE partner repository 81 with the dataset identifier PXD609575 and 10.6019/PXD036437.

Human research participants

Policy information about studies involving human research participants and Sex and Gender in Research.

Reporting on sex and gender	NA
Population characteristics	NA
Recruitment	NA
Ethics oversight	NA

Note that full information on the approval of the study protocol must also be provided in the manuscript.

Field-specific reporting

Please select the one below that is the best fit for your research. If you are not sure, read the appropriate sections before making your selection.

☒ Life sciences ☐ Behavioural & social sciences ☐ Ecological, evolutionary & environmental sciences

For a reference copy of the document with all sections, see nature.com/documents/nr-reporting-summary-flat.pdf

Life sciences study design

All studies must disclose on these points even when the disclosure is negative.

Sample size	Number of animals was determined a priori using the GPower software based on our previous publication (PMID: 34428340) or pilot experiments.
Data exclusions	No data was excluded.
Replication	Key findings were reproducible by two independent researchers.
Randomization	Animals were housed with 3 female fish per tank to avoid social isolation which influences brain plasticity. Tanks were randomly assigned to control and experimental groups.
Blinding	Investigators were blinded during analysis.

Reporting for specific materials, systems and methods

We require information from authors about some types of materials, experimental systems and methods used in many studies. Here, indicate whether each material, system or method listed is relevant to your study. If you are not sure if a list item applies to your research, read the appropriate section before selecting a response.

Materials & experimental systems

n/a	Involved in the study
☐	☒ Antibodies
☒	☐ Eukaryotic cell lines
☒	☐ Palaeontology and archaeology
☐	☒ Animals and other organisms
☒	☐ Clinical data
☒	☐ Dual use research of concern

Methods

n/a	Involved in the study
☒	☐ ChIP-seq
☒	☐ Flow cytometry
☒	☐ MRI-based neuroimaging

Antibodies

Antibodies used	rabbit anti-L-plastin (1/500, Sigma-Aldrich, SAB2701743), rabbit anti-SOX2 (1/1000, Sigma-Aldrich, SAB2701800), mouse anti-HuC/D (1/200, Thermo Fisher, A-21271), mouse anti-PCNA (1/500, Abcam, ab29), Goat anti-BLBP (1/1000, Abcam, ab110099), rat anti-BrdU (1/1000, Abcam, ab6326), and mouse anti-GS (1/1000, Abcam, ab64613). We performed anti-WFA staining with a biotinylated Lectin from Wisteria Floribunda (WFA, 1:500, L1516, Sigma-Aldrich). TUNEL labelling was performed according to the manufacturer guidelines (Sigma-Aldrich, 11684795910).

Validation	All antibodies have been previously used on killifish (PMID: 34428340). Since killifish is a non-canonical research model, killifish-specific antibodies are non-existing. Due to the close evolutionary distance with zebrafish (Danio rerio), zebrafish-specific antibodies often do have specificity for killifish. Whenever available on the producers' website (or via personal communication with the producer), the immunogen of the antibody was aligned via pBLAST to the killifish NCBI database. Antibodies were only used if an alignment of more than 70% could be reached. All staining patterns observed were compared with staining patterns in the zebrafish brain. Negative control samples were always included.

Animals and other research organisms

Policy information about studies involving animals; ARRIVE guidelines recommended for reporting animal research, and Sex and Gender in Research

Laboratory animals	All experiments were performed on adult, young and aged (6 week- and 17 till 18 week-old) female African turquoise killifish (Nothobranchius furzeri, strain GRZ-AD)
Wild animals	NA
Reporting on sex	All experiments were performed on female killifish. Sex was determined based on coloration (killifish are sexual dimorph teleosts).
Field-collected samples	NA
Ethics oversight	All experiments were approved by the KU Leuven ethical committee in accordance with the European Communities Council Directive of 22 September 2010 (2010/63/EU) and the Belgian legislation (KB of 29 May 2013).

Note that full information on the approval of the study protocol must also be provided in the manuscript.
